# Data supporting exosome laden oxygen releasing antioxidant and antibacterial cryogel wound dressing OxOBand alleviate diabetic and infectious wound healing

**DOI:** 10.1016/j.dib.2020.105671

**Published:** 2020-05-11

**Authors:** Parvaiz A. Shiekh, Anamika Singh, Ashok Kumar

**Affiliations:** a Biomaterial and Tissue Engineering Group, Department of Biological Sciences and Bioengineering, Indian Institute of Technology Kanpur, Kanpur, India; b Centre for Nanosciences, Indian Institute of Technology Kanpur, Kanpur, India; c Centre for Environmental Sciences and Engineering, Indian Institute of Technology Kanpur, Kanpur, India

**Keywords:** Diabetic wounds, Antioxidant biomaterials, Oxygen releasing scaffolds, Exosomes, Cryogels

## Abstract

Hypoxia, reduced vascularization, elevated oxidative stress, and infection are critical clinical hallmarks of non-healing chronic diabetic wounds. The dataset presented here is in support of the development and evaluation of the exosome laden oxygen releasing OxOBand for treatment and management of diabetic and infectious wounds [1]. It describes the additional results in support of the development of OxOBand and its evaluation for diabetic wound healing. Exosomes were isolated from adipose-derived stem cells (ADSCs) and characterized through dynamic light scattering (DLS) and scanning electron microscopy (SEM). The encapsulation of exosomes by cells and its effect on migration of NIH3T3 cells under in-vitro condition is described. Further antioxidant polyurethane (PUAO) cryogel and oxygen releasing antioxidant (PUAO-CPO) cryogel scaffolds were fabricated as reported earlier [2,3] and NIH3T3, HaCaT and ADSCs were cultured on these scaffolds. “OxOBand”, the calcium peroxide containing oxygen releasing antioxidant polyurethane (PUAO-CPO) scaffold along with exosomes was evaluated in chronic wounds in diabetic rats. The wounds were also infected with *Staphylococcus aureus (S. aureus)*, and *Pseudomonas aeruginosa (P. aeruginosa)* bacteria and OxOBand was further evaluated for the healing of these infectious diabetic wounds. Interpretation of this data can be found in a research article title “Exosome laden oxygen releasing antioxidant and antibacterial cryogel wound dressing OxOBand alleviate diabetic and infectious wound healing, Shiekh et. al., Biomaterials, 2020 [1].

**Specifications Table**

**Table:** 

Subject	Materials Science, Biomaterials:
Specific subject area	The development and evaluation of oxygen releasing antioxidant dressing for treatment of diabetic ulcers
Type of data	Graphs and Figures
How data were acquired	The data was acquired using dynamic light scattering (DLS), scanning electron microscopy (SEM), transmission electron microscopy (TEM), fluorescent microscopy and bright field microscopy
Data format	Raw data including microscopic images Analyzed data including histograms and graphs
Parameters for data collection	The data was collected in in-vitro conditions using cell lines and primary cells and under in-vivo conditions using diabetic wound model in rats
Description of data collection	The data was collected using various assays such as live/dead cell integrity assay, microscopic imaging, MTT assay and bacterial culture
Data source location	Indian Institute of Technology Kanpur, Kanpur, India
Data accessibility	Data is available from the corresponding author upon reasonable request and **mendeley data with** data identification URL: https://data.mendeley.com/datasets/92 × 2k57dj8/draft?a=5b5aeb28-2eb7-4ac6-a650-39b4a3889272
Related research article	P.A. Shiekh, A. Singh, A. Kumar, Exosome laden oxygen releasing antioxidant and antibacterial cryogel wound dressing OxOBand alleviate diabetic and infectious wound healing, Biomaterials. 249 (2020) 120020

## Value of the data

• The data further supports the role of oxygen and antioxidant scaffolds in diabetic wound healing
• The dataset also presents the growth and proliferation of fibroblasts, keratinocytes and adipose- derived stem cells on PUAO and PUAO-CPO scaffolds
• The data is beneficial for the researchers in the field of biomedical engineering, basic biologists as well as the clinicians to understand and develop better treatment strategies for diabetic ulcers

## 1 Data Description

**Fig. 1:**
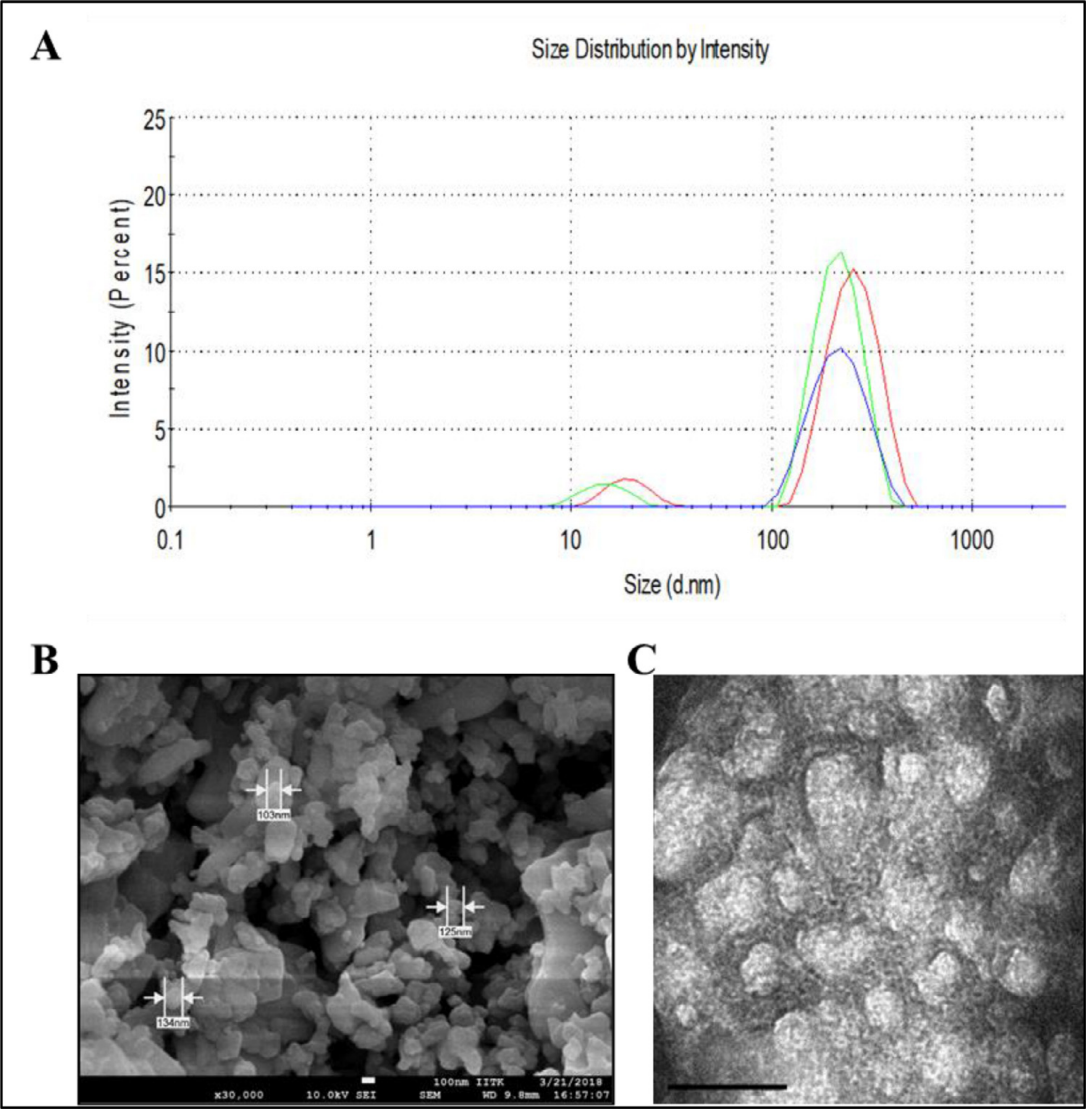
(A) Dynamic light scattering (DLS) analysis of adipose-derived stem cell (ADSC) exosomes are showing the average size and distribution. (B) Scanning electron microscopic images of ADSC derived exosomes showing the circular and cup-shaped morphology. (C) Transmission electron microscopic images of ADSC derived exosomes.

**Fig. 2:**
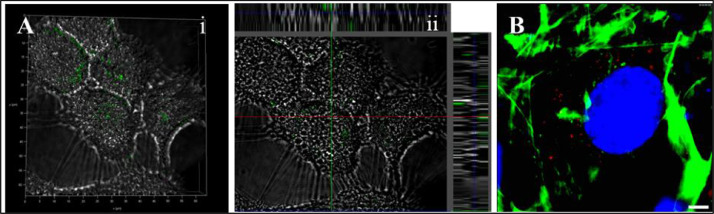
Uptake of adipose-derived stem cell (ADSC) exosomes by HaCaT and NIH3T3 cells. (A) (i) 3D confocal microscopic images showing that the exosomes were internalised intact within the HaCaT cells and are mostly concentrated around the nucleus. (ii) The orthogonal sections in confocal microscopy indicated that the exosomes were localized inside the cells in the cytoplasm. (B) Confocal laser scanning image of NIH3T3 cells showing endocytosis of PKH26 (red) labelled exosomes. Exosomes were labelled with PKH26 dye and incubated with cells for 12 h. Cells were stained with FITC-Phalloidin and DAPI for cytoskeleton and nuclear visualization. scale bar: 3 µm

**Fig. 3:**
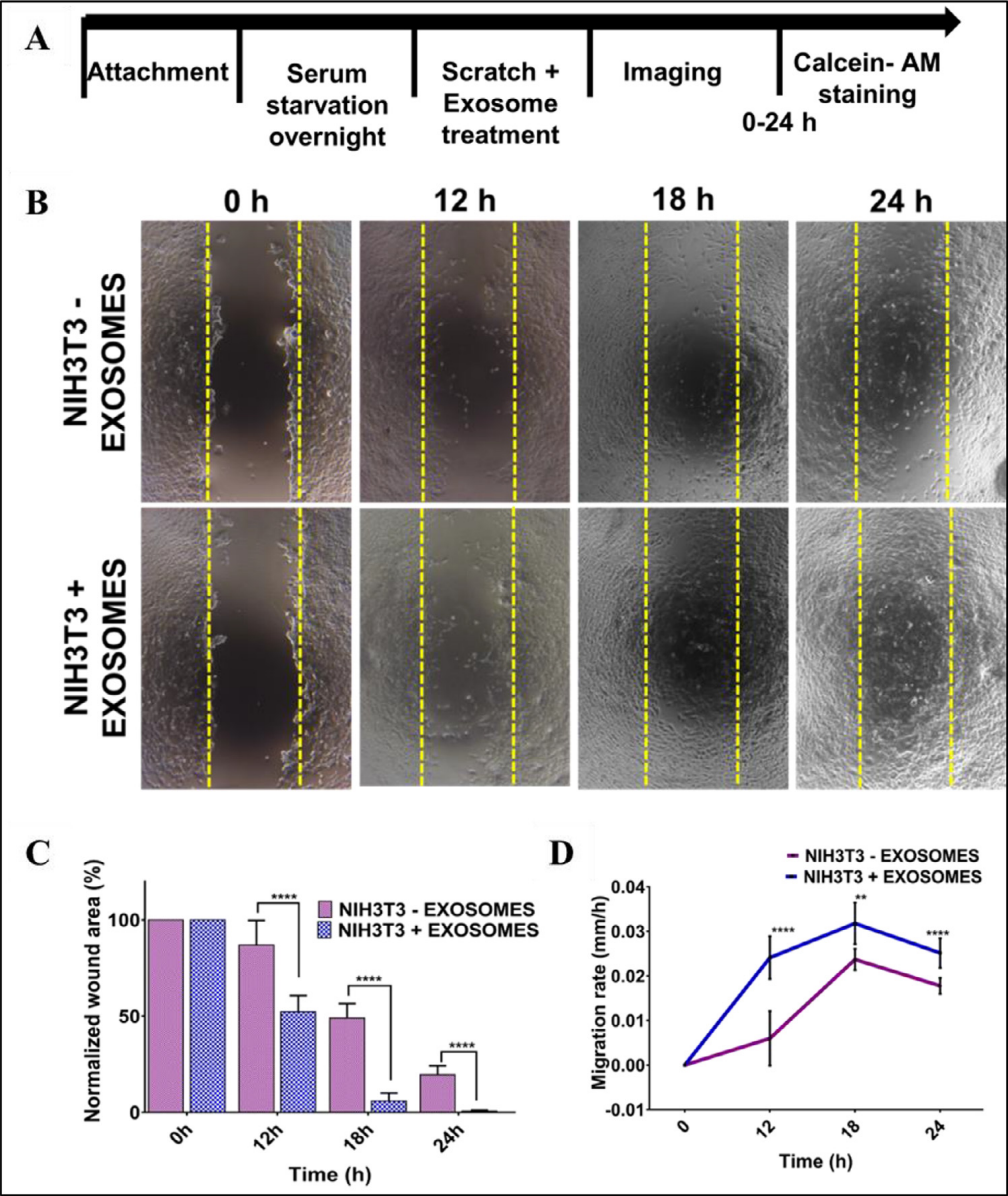
Adipose-derived stem cell (ADSC) exosomes enhanced the migration of NIH3T3 fibroblasts. (A) Experimental timeline. Cells were seeded in a multi-well plate. After reaching confluency, cells were serum-starved overnight before wounding and exosome treatment. Cells were imaged at different time intervals upto 24 h. (B) Exosome treatment enhanced fibroblast migration. Representative images are showing wound closure after 12 h, 18 h and 24 h. The wound was closed in 24 h. (C) Histogram showing wound area covered in fibroblast cells on treatment with exosomes as compared to control. (D) The migration rate of NIH3T3 cells upon exosome treatment. Cells showed an increased migration rate on exosome treatment. **p ≤ 0.01, ***p ≤ 0.001, ****p ≤ 0.0001

**Fig. 4:**
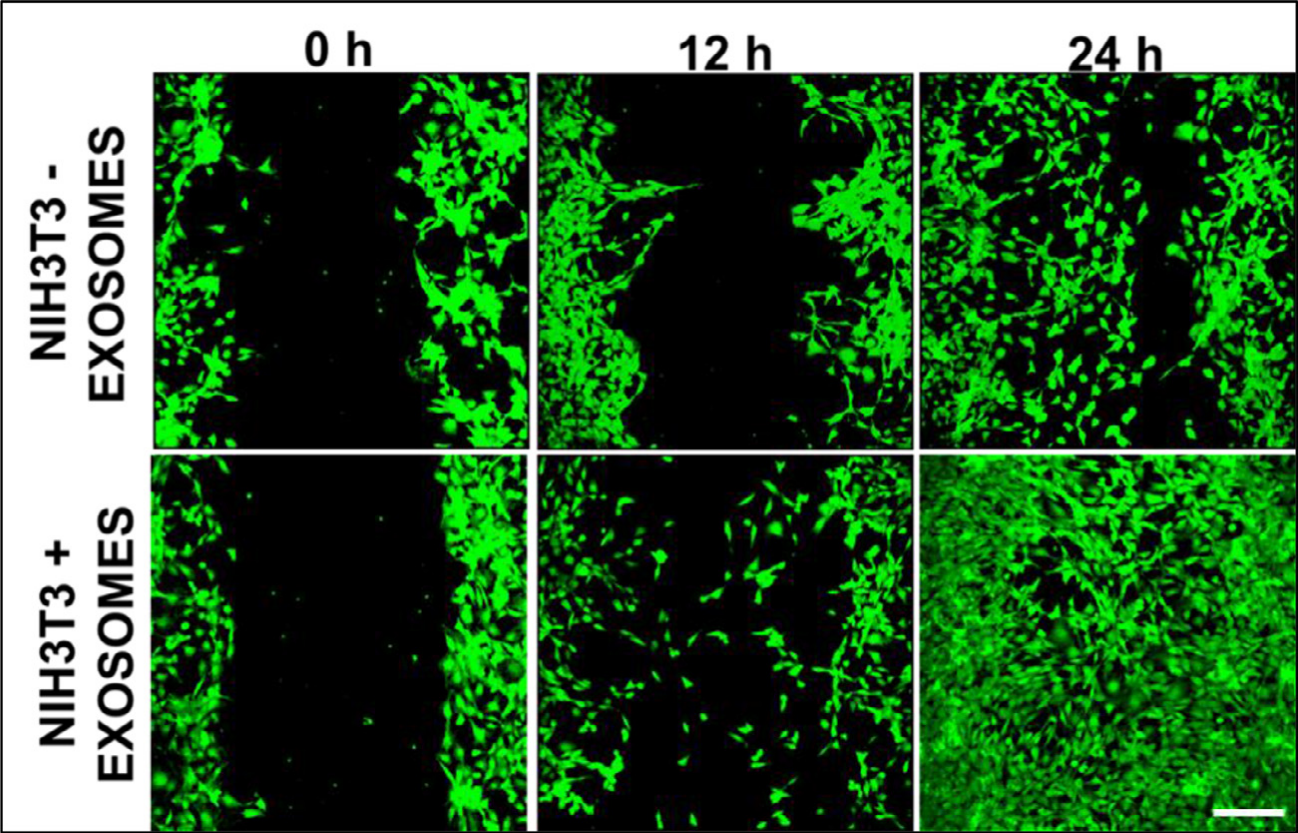
Calcein AM stained live-cell imaging showing wound closure of fibroblasts at different time points (0 h, 12 h, 24 h). Wound closure was mainly due to migration as the cell density was almost similar in both exosome treated and control groups. Scale bar: 200 µm

**Fig. 5:**
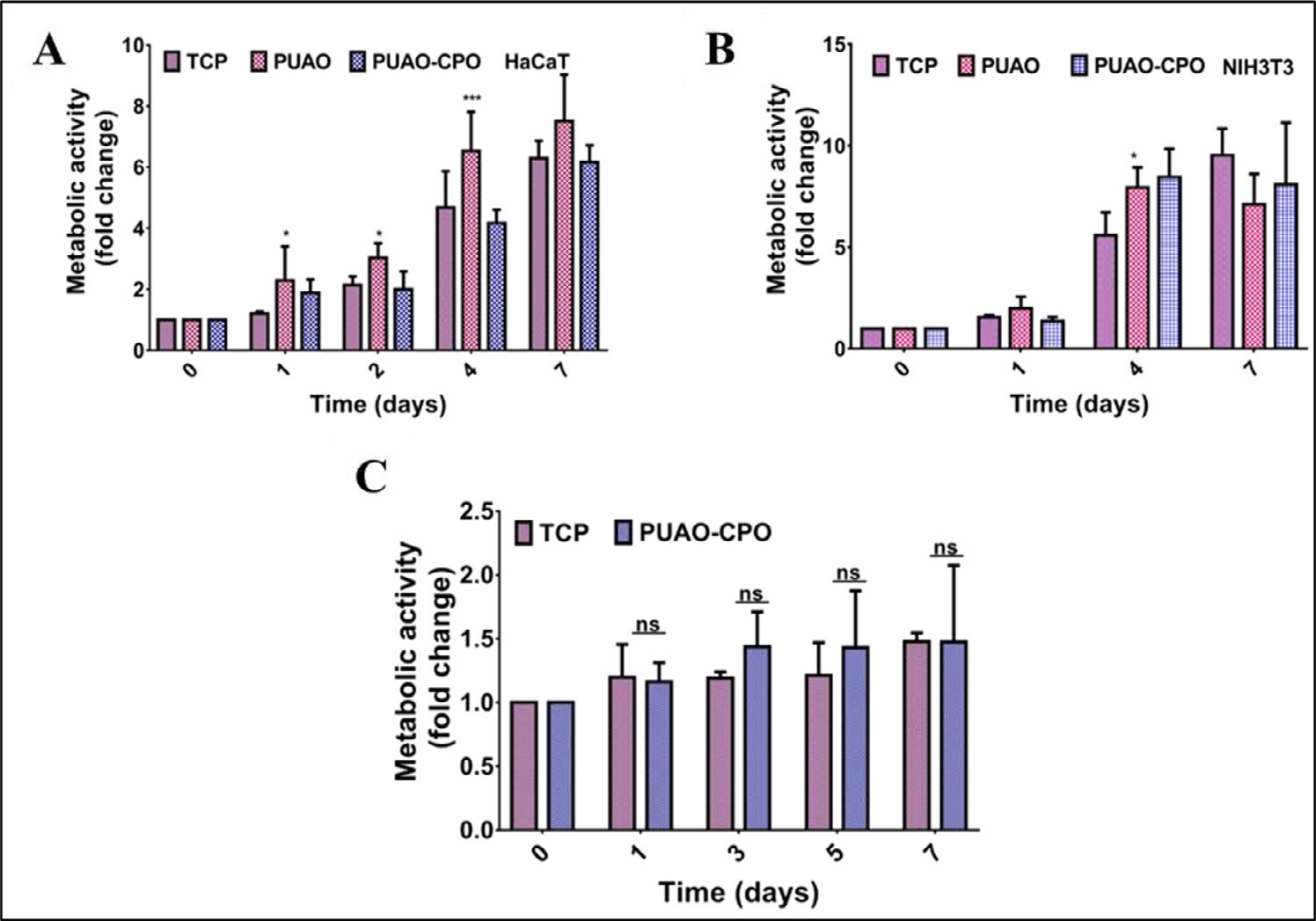
Cytocompatibility of antioxidant polyurethane (PUAO) and calcium peroxide containing oxygen releasing antioxidant polyurethane (PUAO-CPO) scaffolds. (A) Metabolic activity of HaCaT keratinocytes on PUAO and PUAO-CPO scaffolds. Cells are compatible with both scaffolds and showed enhanced proliferation for 7 days. Interestingly HaCaT showed significantly decreased proliferation on oxygen releasing scaffold than antioxidant scaffold, which may indicate its role in cell migration. (B) Metabolic activity of NIH3T3 fibroblasts on PUAO and PUAO-CPO scaffolds. Cells showed enhanced proliferation on antioxidant and oxygen releasing scaffolds for 7 days. (C) Metabolic activity of ADSCs on PUAO-CPO scaffolds. The cells showed enhanced proliferation on PUAO-CPO scaffolds cultured for 7 days. *p≤0.05. ***p≤0.01, ns: non-significant.

**Fig. 6:**
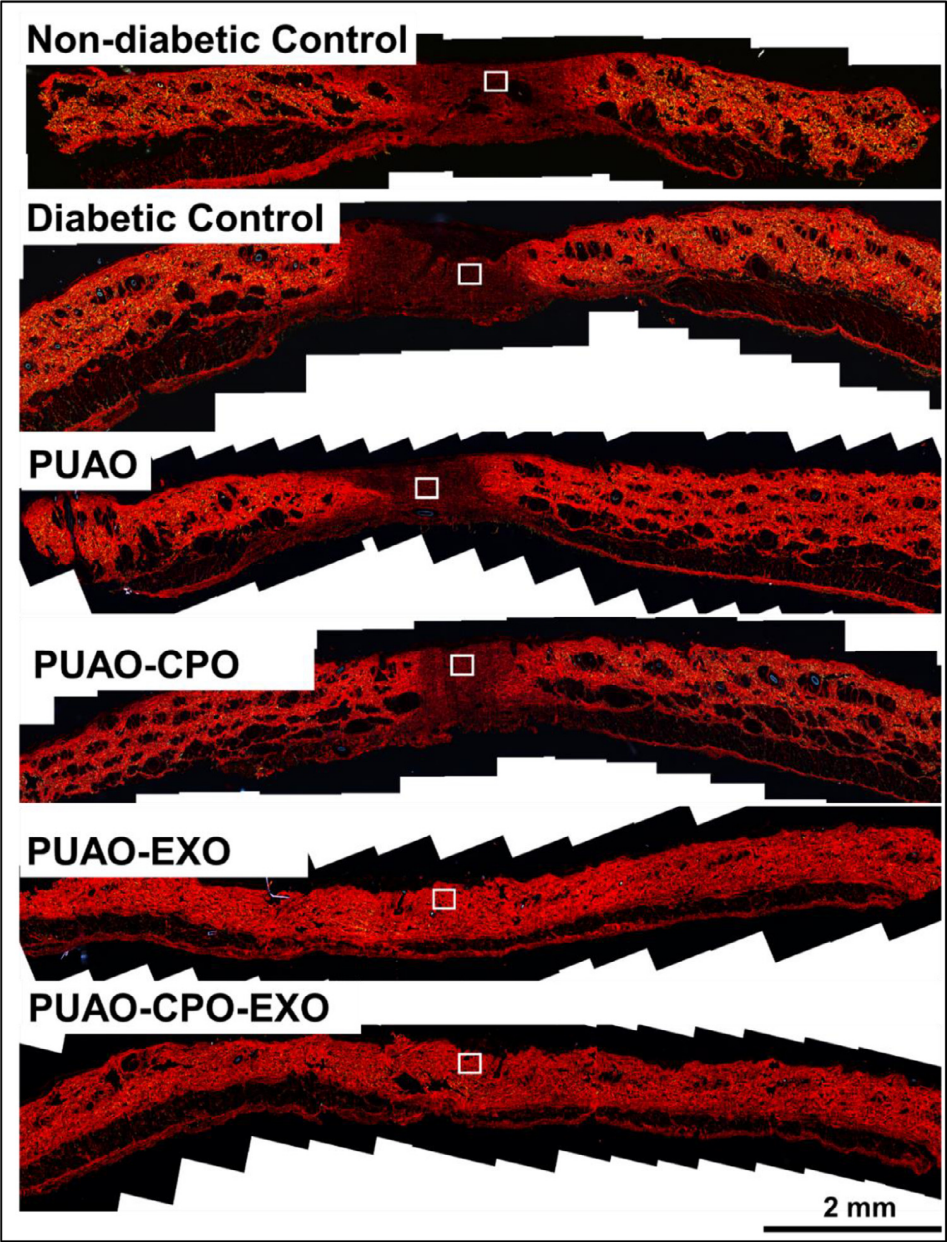
Picrosirius red staining of the whole wound area showing collagen remodeling after oxygen releasing antioxidant scaffold and exosome treatment.

**Fig. 7:**
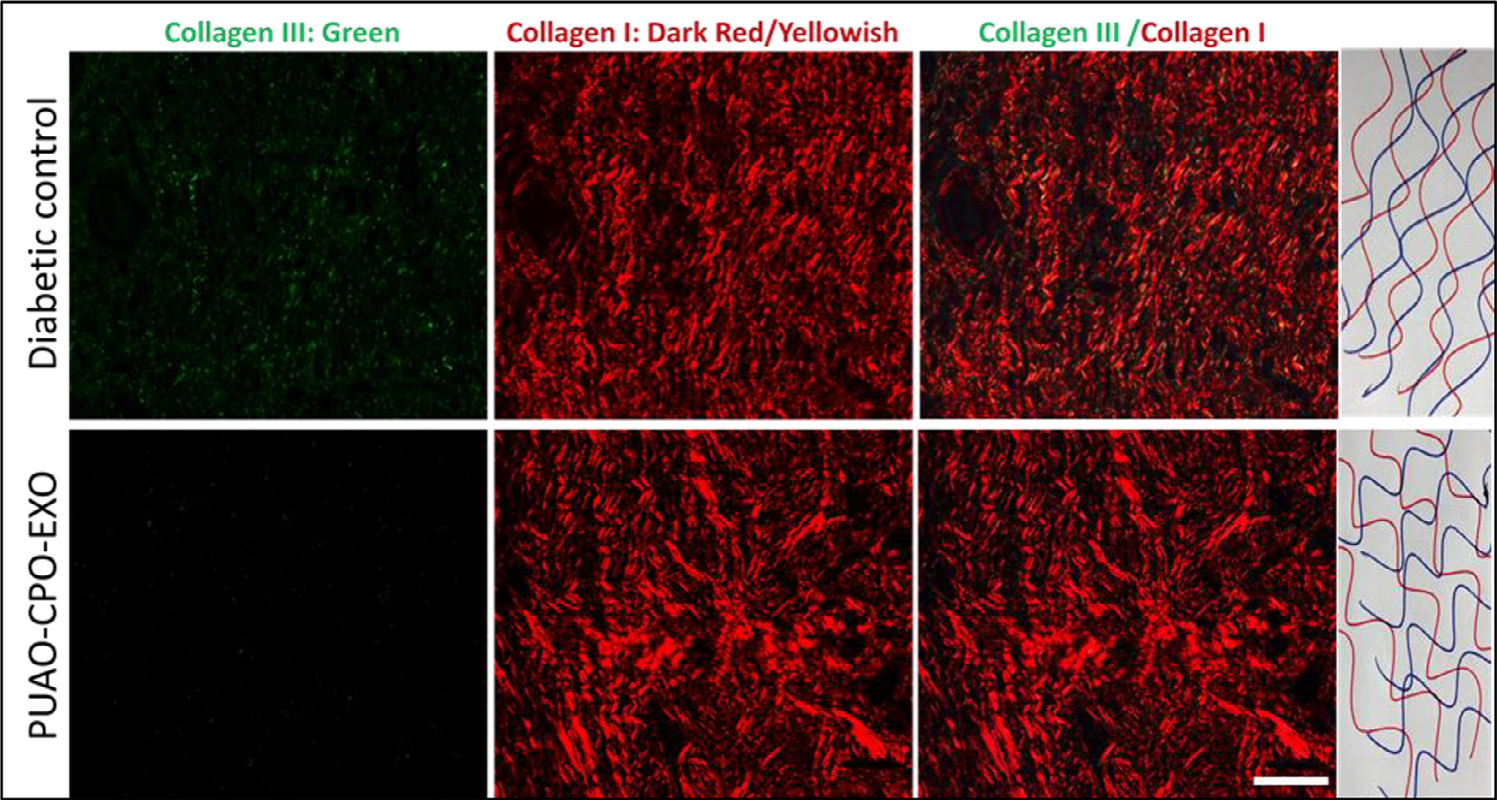
Deposition of collagen I and collagen III in calcium peroxide containing oxygen releasing antioxidant polyurethane scaffolds along with exosomes (PUAO-CPO-EXO) treated wounds. The collagen has a native basketweave structure in PUAO-CPO-EXO treated groups. Scale bar: 200 µm

**Fig. 8:**
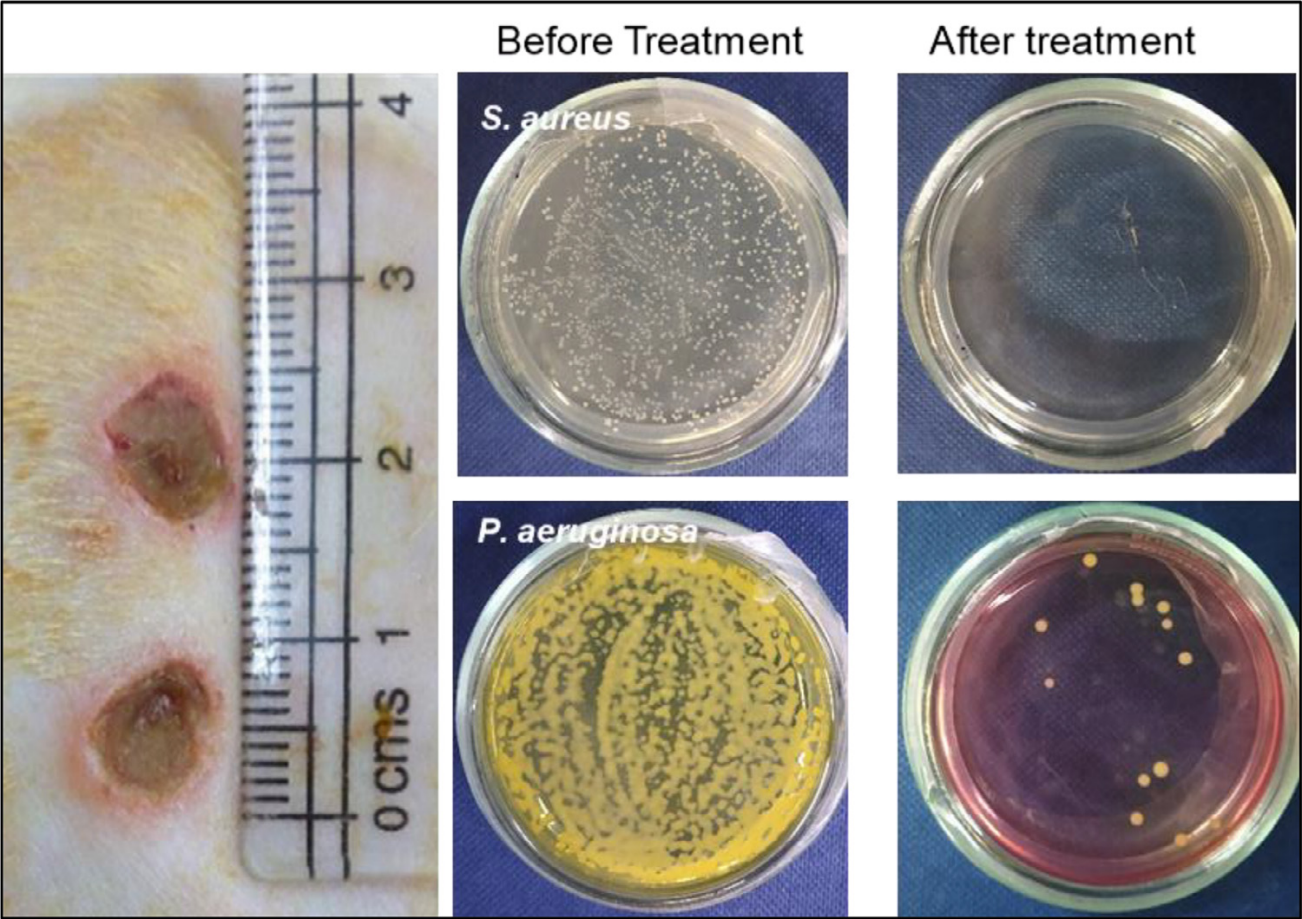
Development of infection in diabetic wounds after 3 days of inoculation of *Staphylococcus aureus* (*S. aureus*), and *Pseudomonas aeruginosa* (*P. aeruginosa*) in a ratio of 1:1. Bacterial count before and after treatment with calcium peroxide containing oxygen releasing antioxidant polyurethane scaffold along with exosomes (PUAO-CPO-EXO).

The dataset reported in this article describes the data in support of the development of exosome laden oxygen releasing wound dressing for diabetic wound healing. Fig. 1 describes the characterisation of adipose-derived stem cell (ADSC) exosomes through dynamic light scattering (DLS), scanning electron microscopy (SEM) and transmission electron microscopy (TEM). Fig. 2 describes the encapsulation of adipose-derived stem cell (ADSC) exosomes by HaCaT and NIH3T3 cells. Fig. 3 describes the effect of adipose-derived stem cell (ADSC) exosomes on the migration of NIH3T3 fibroblasts under in-vitro conditions. Fig. 4 depicts the Calcein AM stained live-cell imaging showing wound closure of NIH3T3 fibroblast cells upon exosome treatment. Fig. 5 represents the metabolic activity of HaCaT keratinocytes, NIH3T3 fibroblasts and adipose-derived stem cells (ADSCs) on PUAO and PUAO-CPO scaffolds. Fig. 6 illustrates picrosirius red staining of the diabetic wound area showing collagen remodeling after oxygen releasing antioxidant scaffold and exosome treatment. Fig. 7 describes the deposition of collagen I and collagen III in oxygen releasing antioxidant polyurethane scaffolds along with exosomes (PUAO-CPO-EXO) treated wounds. Fig. 8 illustrates the development of infection in diabetic wounds and its decline after PUAO-CPO-EXO dressing application.

## 2 Experimental Design, Materials, and Methods

Here, we describe the characterisation of exosomes isolated from adipose-derived stem cells (ADSCs). The isolated exosomes were evaluated for their cellular encapsulation, and effect on cellular migration. We also describe the evaluation of antioxidant polyurethane (PUAO) and oxygen releasing antioxidant polyurethane (PUAO-CPO) cryogel scaffolds for growth and proliferation of keratinocytes (HaCaT), fibroblasts (NIH3T3) and adipose-derived stem cells (ADSCs). Data supporting the healing and collagen remodelling in diabetic wounds on treatment with OxOBand, the calcium peroxide containing antioxidant polyurethane (PUAO-CPO) scaffold along with exosomes (PUAO-CPO-EXO) is described here. Development of infection and its treatment with OxOBand is also reported.

### 2.1 Characterisation of exosomes

Exosomes were characterised for size distribution through dynamic light scattering (DLS) analysis. Briefly, exosome samples were diluted in millQ water and filtered through 0.22 µm filter, loaded into the cuvette and readings were obtained using Zetasizer (Malvern Panalytical Ltd, NL). Scanning electron microscopy (SEM) and transmission electron microscopy (TEM) was carried out for morphological analysis of the exosomes. For SEM analysis, exosomes were fixed in 4% paraformaldehyde (PFA), washed with milliQ water and dried in a desiccator before imaging. The images were obtained at an accelerating voltage of 10 kV using FEI-Quanta 200. For TEM analysis, exosomes were fixed in 1% glutaraldehyde, counterstained with 1% uranyl acetate, and imaged at an accelerating voltage of 120 kV using FEI-Tecnai G2.

### 2.2 Exosome uptake and internalisation

Exosome uptake was studied in HaCaT and NIH3T3 cells. Internalisation of intact exosomes was studied in HaCaT cells by labelling them with Calcein AM stain. Calcein AM is a membrane permeable dye, which shows fluorescence once inside the exosomes due to esterase activity. The images were acquired using confocal microscopy (Leica SPE II, Germany). Uptake was also studied in NIH3T3 cells by labelling exosomes with PKH26 dye (Sigma Aldrich, USA). For nuclear and cytoskeleton visualisation, cells were stained with DAPI and Phalliodin-FITC dye, respectively. The images were acquired using confocal microscopy (Leica SPE II, Germany).

### 2.3 Cell migration in presence of exosomes

The effect of ADSCs derived exosomes on cellular migration was studied in NIH3T3 fibroblast cells. Cells were cultured in a 24 well tissue culture plate for 24 h. To reduce the cell proliferation, cells were serum-starved overnight. A micro scratch in the cell layer was made at the centre of 24 wells plate, cell debris was washed and treated with exosomes at a concentration of 20 µg/ml. Cells without any exosome treatment were taken as control. The migration was monitored for 24 h and images were acquired at different time intervals. From the acquired images, wound closure percentage was calculated through ImageJ software. The area of the wound remaining at each time interval was calculated and normalised with the area just after scratch. From the wound area, the migration rate was calculated by dividing the area of migration by time elapsed. For live-cell imaging and visualisation, cells were also stained with Calcein AM dye and imaged at different time intervals.

### 2.4 Cell culture studies on PUAO and PUAO-CPO cryogel scaffolds

For cell culture studies, PUAO and PUAO-CPO scaffolds were sterilised using gradient ethanol. Briefly, cryogels were washed with 70% ethanol for 1 h, followed by 100% ethanol under UV light for 1 h. The cryogel scaffolds were further washed three times with 1x phosphate buffer saline (PBS) and incubated in Dulbecco's modified eagle medium (DMEM) + 10% fetal bovine serum (FBS) overnight. Next day, cells were trypsinized and counted using a haemocytometer. On each scaffold, 5 × 10^4^ cells were seeded in 20 µl DMEM and incubated for 4 h for cell attachment in a 48 well plate. After 4 h, 200 µl of media containing 10% FBS was added. HaCaT, NIH3T3 and adipose-derived stem cells (ADSCs) were cultured on PUAO and PUAO-CPO cryogel scaffolds. Cells cultured on tissue culture plate (TCP) were used as control. Metabolic activity was assayed using MTT assay at different time intervals. Briefly, media was aspirated from the wells and 200 µl of MTT was added. After 4 h incubation at 37°C, MTT was aspirated and the formazan crystals formed were dissolved in DMSO by incubation at 37°C for 15 min. Metabolic activity was determined by measuring absorbance at 570 nm in a microplate reader (BioTek, USA). All values were normalized with day 0 and represented as mean ± s.d.

### 2.5 Collagen remodelling by picrosirius red staining

For collagen remodelling analysis, animal tissues were stained with picrosirius red. Briefly animal tissues after harvesting were fixed in 10% formalin in PBS for 72 h. The fixed tissues were dehydrated in gradient ethanol, embedded in paraffin wax, sectioned into 5 µm sections using microtome (Leica, Germany) and collected on super frost slides coated with poly-L-lysine. The sections were stained with picrosirius red stain and imaged under polarised light microscopy using Leica DM 2500 microscope. The remodelling of collagen was analysed by ImageJ software.

### 2.6 Infection studies in diabetic wounds

For infection studies in diabetic wounds, the diabetic wounds were infected with *Staphylococcus aureus* (*S. aureus*), and *Pseudomonas aeruginosa* (*P. aeruginosa*) in a ratio of 1:1. Briefly, diabetes was induced in rats by feeding with high fat diet followed by intraperitoneal injection of two alternate doses of streptozocin (30 mg/kg body weight). Four weeks after diabetic induction, animals with glucose level above 250 mg/dl were used for further experimentation. Two 8 mm wounds were created at the back of rat and inoculated with 1 × 10^8^ CFU/ml of *Staphylococcus aureus* (*S. aureus*), and *Pseudomonas aeruginosa* (*P. aeruginosa*) in a ratio of 1:1. Three days after ulcer formation, bacterial swabs were collected using q tip and cultured in specific media for *Staphylococcus aureus* (S. aureus), and *Pseudomonas aeruginosa* (P. aeruginosa) to calculate CFUs. The wounds were treated with PUAO-CPO scaffolds along with exosomes for 21 days. Bacterial CFUs were also calculated after 21 days of treatment to analyse control in infection.

### 2.7 Statistical analysis

Statistical analysis was carried out using Graphpad Prism 7.0. A statistical difference of *p≤0.05* was considered significant.

## Declaration of Competing Interest

The authors declare that they have no known competing financial interests or personal relationships which have, or could be perceived to have, influenced the work reported in this article.

## References

[1] Shiekh P.A., Singh A., Kumar A. (2020). Exosome laden oxygen releasing antioxidant and antibacterial cryogel wound dressing OxOBand alleviate diabetic and infectious wound healing. Biomaterials.

[2] Shiekh P.A., Singh A., Kumar A. (2018). Engineering Bioinspired Antioxidant Materials Promoting Cardiomyocyte Functionality and Maturation for Tissue Engineering Application. ACS Appl. Mater. Interfaces..

[3] Shiekh P.A., Singh A., Kumar A. (2018). Oxygen-Releasing Antioxidant Cryogel Scaffolds with Sustained Oxygen Delivery for Tissue Engineering Applications. ACS Appl. Mater. Interfaces..

